# Dataset for the climate-related financial policy index (CRFPI)

**DOI:** 10.1016/j.dib.2023.109044

**Published:** 2023-03-08

**Authors:** Paola D'Orazio

**Affiliations:** Chair of Macroeconomics, Faculty of Management and Economics, Ruhr-Universität Bochum, Universitätsstraße 150, 44801 Bochum, Germany

**Keywords:** Climate-related financial policies, Climate risks, Low-carbon transition, Financial stability, Composite index, Green central banking, Prudential regulations, Green bonds

## Abstract

Data on the climate-related financial policy index (CRFPI) - comprising the global climate-related financial policies adopted globally and the bindingness of the policy - are provided in this paper for 74 countries from 2000 to 2020.

The data include the index values from four statistical models used to calculate the composite index as described in [3]. The four alternative statistical approaches were designed to experiment with alternative weighting assumptions and illustrate how sensitive the proposed index is to changes in the steps followed to construct it.

The index data shed light on countries’ engagement in climate-related financial planning and highlight policy gaps in relevant policy sectors. Researchers could use the data provided in this paper to study green financial policies further and compare countries, highlighting engagement in specific policy areas or the entire spectrum of climate-related finance policy. Moreover, the data might be used to investigate the relationship between green finance policy adoption and credit market changes and assess their effectiveness in managing credit and financial cycles in the face of climate risks.

**Specifications Table**

**Table:** 

Subject	Finance and Banking
Specific subject area	Climate-related financial policy and green central banking
Type of data	Table (Excel file)
How the data were acquired	Data on policies was acquired from the CRFPdata: 2000-2020 data repository [2].
Data format	Analyzed
Description of data collection	Data contained in this article were generated by applying the statistical method for developing composite indices described in [1]. Data collected from the CRFPdata: 2000-2020 data repository [2] were classified into five categories based on existing literature and taxonomy. A policy indicator that considers each policy category and its bindingness is computed and normalized using the min–max method. For each country and each year considered in the analysis (2000-2020), the individual rescaled policy indicators are aggregated in a composite index (i.e., the CRFPI) by following a statistical aggregation method based on additive weighting.
Data source location	Primary data available at the CRFPdata: 2000-2020 data repository [2]. 74 countries, of which: ■ ***39 advanced economies***: Australia Austria Bahrain Belgium Canada, Chile, Croatia, Cyprus, Czech Republic, Denmark, Finland, France, Germany, Greece, Hungary, Iceland, Ireland, Israel, Italy, Japan, Latvia, Lithuania, Luxembourg, Netherlands, New Zealand, Norway, Panama, Poland, Portugal, Saudi Arabia, Seychelles, Singapore, South Korea, Spain, Sweden, Switzerland, United Arab Emirates, United Kingdom, United States of America ■ ***20 emerging economies***: Argentina, Brazil, Bulgaria, China, Colombia, Costa Rica, Ecuador, Fiji, Georgia, Indonesia, Kazakhstan, Lebanon, Malaysia, Mexico, Paraguay, Peru, Russia, South Africa, Thailand, Turkey ■ ***15 developing countries***: Bangladesh, Cambodia, Egypt, Ghana, India, Kenya, Mongolia, Morocco, Nepal, Nigeria, Pakistan, Philippines, Sri Lanka, Ukraine, Viet Nam.
Data accessibility	Repository name: Zenodo Data identification number: 10.5281/zenodo.7599914 Direct URL to data: https://zenodo.org/record/7599914#.Y9vNCi0w004
Related research article	*D'Orazio, P., & Thole, S. (2022). Climate-related financial policy index: a composite index to compare the engagement in green financial policymaking at the global level. Ecological Indicators, 141, 109065.*https://doi.org/10.1016/j.ecolind.2022.109065

## Value of the Data

• Data contained in this article provide harmonized information on climate-related financial policies at the international level.
• Data on the climate-related financial policy index from 2000 to 2020 are useful as they allow researchers to assess, quantify, and compare international engagement in climate-related financial policymaking.
• Researchers working on green central banking and green finance can benefit from using the data provided in this article to understand the adoption patterns of green financial policies or test empirical relationships by combining the provided data with other data sources.

## 1 Objective

Central banks and financial regulators' policies cannot replace climate policies, but it is widely acknowledged that they must promote green financing and develop regulations for climate-related financial risks [3], [4], [5], [6]. This is because climate change impacts monetary policy and financial regulation [6,7], and financial actors are crucial in the global economy [8,9].

International involvement in climate finance policy-making has increased in recent years, albeit to varying degrees [10]. However, comparing countries' performance globally is challenging as different criteria are often used to evaluate the “greenness” of a financial system, central bank, or financial regulator. The composite index CRFPI is based on a statistical methodology that involves normalizing, weighting, and aggregating five components (climate-related financial policy types) [7]. This approach creates an objective scoring system for climate finance policies, eliminating subjective assessments of specific policies' performance or significance.

The data provided in this article allow researchers and policymakers to evaluate global engagement in climate-related financial policies. Moreover, they could be used to replicate the findings in [1] and perform additional analysis on the role played by climate-related financial policies in the past 20 years. Additionally, the data might be used to investigate the relationship between green finance policy adoption and credit market changes and assess their effectiveness in managing credit and financial cycles in the face of climate risks.

## 2 Data Description

The repository [11] contains four excel files, each referring to a statistical methodology used to calculate the composite CRFP index. The content of the files and the corresponding methodology are summarized in Table 1.

**Table 1:** Overview of the content of the repository: composite indices and their aggregation and weighting methods.

File name	Name of the index	Method for composite index computation	Method description
CRFPI1_dataset_v1.0.xlsx	CRFPI1	Additive model with equal weights for all policy categories	Weighted sum of policies *with* policy bindingness
CRFPI2_dataset_v1.0.xlsx	CRFPI2	Additive model with different weights for policy categories	Weighted sum of policies *with* bindingness and differentiated weights for the prudential (GPP) and credit allocation (GCA) policy areas
CRFPI3_dataset_v1.0.xlsx	CRFPI3	Additive model with equal weights for all policy categories	Weighted sum of policies *without* bindingness
CRFPI4_dataset_v1.0.xlsx	CRFPI4	Additive model with equal weights for all policy categories	Weighted sum of policies *with* bindingness and detailed policy indicator for the prudential policy area

## 3 Experimental Design, Materials and Methods

Data collected from the CRFPdata: 2000-2020 data repository [2] were classified into five categories (described below) based on existing literature and taxonomy. Afterward, a policy indicator that considers each policy category and its bindingness is computed and normalized using the min–max method.

**Fig. 1:**
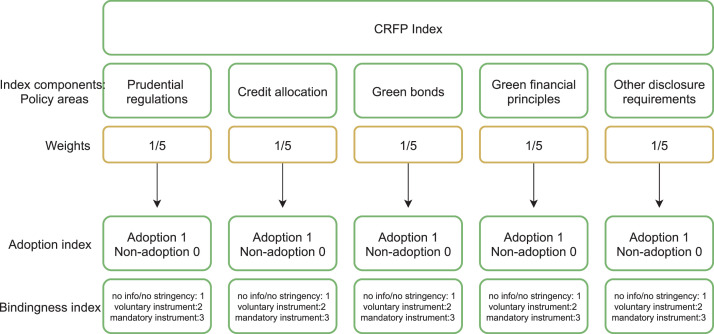
Design of the climate-related financial policy (CRFP) index. Source: [1].

For each country and each year considered in the analysis, the individual rescaled policy indicators are aggregated in a composite index (i.e., the CRFPI) by following a standard statistical-methodological approach for constructing composite indices as described in [1]. An overview of the design of the index is illustrated in Fig. 1.

In particular, the four main steps for constructing the index are as follows:

(i) *definition of the individual five policy indicators*. Data retrieved from the CRFPdata: 2000-2020 data repository [2] were classified into five categories based on existing literature and taxonomy, as follows:
  I. **Policy Area (I) - Green Prudential Regulations (GPP)**
    Measures meant to identify threats to financial stability in the presence of climate-related risks. They are implemented as capital regulations, changes of governance, risk management measures, climate-related stress tests, and climate-related risk disclosure aimed at the banking sector.
  II. **Policy Area (II) - Green Credit Allocation Policies (GCA)**
    Policies promoting green lending and investments through credit allocation such as green lending quotas, concessional loans or priority to environmentally friendly sectors, and lending limits to “brown” sectors.
  III. **Policy Area (III) - Green Financial Guidelines (GFG)**
    To promote green financial markets, it is necessary to determine coherent prin- ciples and produce reliable taxonomies to correctly evaluate the true impact of investments on the environment. Other similar policies concern guidelines for sustainability reporting and compliance practices.
  IV. **Policy Area (IV) - Other Green Disclosure Requirements (OGD)**
    Measures mandating or recommending specific environmental criteria for pension funds, insurance companies, and other non-financial institutions.
  V. **Policy Area (V) - Green Bonds Taxonomy and Issuing (GB)**

Financial assets, such as bonds, are designed to sustain green projects.

(ii) *data normalization.* A policy indicator that considers the policy category and its bindingness is computed and normalized using the min–max method. The normalization considers the upper and lower boundaries within the same period.
(iii) *weighting*. The method involves a simple additive weighting aggregation (SAW) method, according to which the weights are fixed and exogenously set.
(iiii) *aggregation* of the five policy indicators. For each country, the individual rescaled indicators are aggregated in a single index (i.e., the CRFPI) in each period.

Formally, the resulting CRFP index is computed as

$$\text{CRFPI}\left(j,t\right)=\sum_{j=0}^{P}w\;SC\left(j,t\right)$$

where *j* indicates the policy area (as described in point (i) above), *t* refers to the year, *w* is the weight applied (as described in point (iii) above), and *SC* indicates the re-scaled policy indicator (as described in point (ii) above).

## Ethics statements

The authors declare that the present work did not include experiments on human subjects and/or animals.

## CRediT authorship contribution statement

**Paola D'Orazio:** Conceptualization, Data curation, Methodology, Software, Writing – review & editing.

## Declaration of Competing Interest

The author declares no known competing financial interests or personal relationships that could have appeared to influence the work reported in this paper.

## Data Availability

Dataset for the climate-related financial policy index (CRFPI) (v.1.2022) (Original data) (Zenodo).
